# Dovitinib enhances temozolomide efficacy in glioblastoma cells

**DOI:** 10.1002/1878-0261.12076

**Published:** 2017-06-05

**Authors:** Thatchawan Thanasupawat, Suchitra Natarajan, Amy Rommel, Aleksandra Glogowska, Hugo Bergen, Jerry Krcek, Marshall Pitz, Jason Beiko, Sherry Krawitz, Inder M. Verma, Saeid Ghavami, Thomas Klonisch, Sabine Hombach‐Klonisch

**Affiliations:** ^1^ Department of Human Anatomy and Cell Science University of Manitoba Winnipeg Canada; ^2^ Laboratory of Genetics Salk Institute for Biological Studies La Jolla CA USA; ^3^ Department of Surgery University of Manitoba Winnipeg Canada; ^4^ Department of Internal Medicine University of Manitoba Winnipeg Canada; ^5^ Department of Pathology University of Manitoba Winnipeg Canada; ^6^ Department of Medical Microbiology and Infectious Diseases University of Manitoba Winnipeg Canada; ^7^ Obstetrics, Gynecology and Reproductive Medicine College of Medicine University of Manitoba Winnipeg Canada

**Keywords:** DNA damage, dovitinib, glioblastoma, HMGA2, temozolomide

## Abstract

The multikinase inhibitor and FDA‐approved drug dovitinib (Dov) crosses the blood–brain barrier and was recently used as single drug application in clinical trials for GB patients with recurrent disease. The Dov‐mediated molecular mechanisms in GB cells are unknown. We used GB patient cells and cell lines to show that Dov downregulated the stem cell protein Lin28 and its target high‐mobility group protein A2 (HMGA2). The Dov‐induced reduction in pSTAT3^Tyr705^ phosphorylation demonstrated that Dov negatively affects the STAT3/LIN28/Let‐7/HMGA2 regulatory axis in GB cells. Consistent with the known function of LIN28 and HMGA2 in GB self‐renewal, Dov reduced GB tumor sphere formation. Dov treatment also caused the downregulation of key base excision repair factors and O^6^‐methylguanine‐DNA‐methyltransferase (MGMT), which are known to have important roles in the repair of temozolomide (TMZ)‐induced alkylating DNA damage. Combined Dov/TMZ treatment enhanced TMZ‐induced DNA damage as quantified by nuclear γH2AX foci and comet assays, and increased GB cell apoptosis. Pretreatment of GB cells with Dov (‘Dov priming’) prior to TMZ treatment reduced GB cell viability independent of p53 status. Sequential treatment involving ‘Dov priming’ and alternating treatment cycles with TMZ and Dov substantially reduced long‐term GB cell survival in MGMT+ patient GB cells. Our results may have immediate clinical implications to improve TMZ response in patients with LIN28^+^/HMGA2^+^ GB, independent of their MGMT methylation status.

**Abbreviations**
APE1: Apurinic/apyrimidinic endonuclease 1
BBB: Blood–brain barrier
BER: base excision repair
DNA: Deoxyribonucleic acid
Dov: Dovitinib
ES: Embryonic stem cells
FBS: Fetal bovine serum
FEN1: Flap structure‐specific endonuclease 1
FGFR: Fibroblast growth factor receptor
GB: Glioblastoma
HMGA2: High‐mobility group protein A2
IHC: Immunohistochemistry
KD: Knockdown
MGMT: O6‐methylguanine‐DNA methyltransferase
MMS: Methyl methanesulfonate
MPG: *N*‐Methylpurine DNA glycosylase
PARP1: Poly(ADP‐ribose) polymerase 1
PCR: polymerase chain reaction
PDGF: Platelet‐derived growth factor
PI: Propidium iodide
PKC: Protein kinase C
RNA: Ribonucleic acid
SHP1: SH2 domain‐containing protein tyrosine phosphatase
STAT3: Signal transducer and activator of transcription 3
TMZ: temozolomide
VEGF: Vascular endothelial cell growth factor
WB: western blot
XRCC1: X‐ray repair cross‐complementing protein 1
γH2AX: phosphorylated histone 2A (Ser 139)

## 1 Introduction

The FDA‐approved oral drug dovitinib (Dov; TKI258, CHIR258; DOV) inhibits the tyrosine kinases FGFR, PDGFR‐β, VEGFR, and c‐KIT, has manageable toxicity, and distributes extensively in tissues (Dubbelman *et al*., 2012). In addition to its multityrosine kinase inhibitory functions, Dov binds to the DNA minor groove and acts as a topoisomerases I and II poison causing DNA damage (Hasinoff *et al*., 2012). Several phase I/II/III clinical trials revealed that when used as monotherapy, Dov showed only moderate overall efficacy in patient in a variety of solid tumors (Angevin *et al*., 2013; Kang *et al*., 2013; Keam *et al*., 2015; Kim *et al*., 2011; Lim *et al*., 2015; Motzer *et al*., 2014; Sarker *et al*., 2008; Scheid *et al*., 2015). Increased sensitivity to Dov was reported in patients with a RET^G207A^ germline variant (Quintela‐Fandino *et al*., 2014) and in BRAF‐mutant metastatic melanoma (Langdon *et al*., 2015), FGFR2‐mutant endometrial cancer cells (Konecny *et al*., 2013), and BCR‐ABL^+^ leukemia cells (Eucker *et al*., 2014). A beneficial role of Dov comes from combinatorial therapies. *In vitro* and mouse xenograft studies demonstrated a significant benefit in using a combined treatment of Dov with platinum compounds in colon cancer (Gaur *et al*., 2014) and with mTOR inhibitors in hepatocellular (Chan *et al*., 2013) and basal cell breast carcinoma (Issa *et al*., 2013). The combination of Dov and mTOR inhibitor was also found effective in two clinical trials with metastatic renal cancer patients (Blesius *et al*., 2013; Escudier *et al*., 2014), whereas a third trial study reported only modest activity in patients with VEGF‐refractory clear cell renal cancer (Powles *et al*., 2014). A combination of Dov and the antiestrogenic drug fulvestrant (ICI‐182.780) showed a significant *in vitro* antiproliferative activity in human endometrial cancer cells (Eritja *et al*., 2014) and promising clinical activity in a phase II trial with hormone receptor‐positive, HER2+ breast cancer patients (Musolino *et al*., 2017).

Highly malignant glioblastoma (GB) constitutes 50–60% of primary brain tumors and has one of the worst five‐year survival rates among all human cancers (Krex *et al*., 2007). The primary chemotherapeutic drug of choice is the DNA‐alkylating agent temozolomide (TMZ). However, fast growth, the ability to bypass drug actions, and inferior local drug concentrations contribute to inevitable recurrences resulting in fatal chemoresistant forms of GB. Encouraged by the ability of DOV to cross the blood–brain barrier (BBB) (Schafer *et al*., 2016), two clinical trials were initiated in Germany [NCT01972750] and the United States [NCT01753713] to determine whether DOV monotherapy can benefit patients with advanced and recurrent glioblastoma (GB). Recently, first results from the German trial demonstrated efficacy in some recurrent GB patients and recommended additional personalized trials (Schafer *et al*., 2016).

The tumor‐specific responses to Dov and the current lack of studies on molecular mechanisms of Dov action in GB pose a challenge to the development of effective personalized therapeutic strategies. In several human tumor models, Dov was shown to inhibit the MAPK, PI3K/AKT/mTOR, STAT3/5, and/or Wnt signaling pathways (Chase *et al*., 2007; Chon *et al*., 2016; Lopes de Menezes *et al*., 2005; Trudel *et al*., 2005; Zang *et al*., 2015). While the tyrosine kinase receptor inhibitory function of Dov frequently coincided with reduced activity of some of these signaling pathways (Lee *et al*., 2005, 2015; Lopes de Menezes *et al*., 2005; Piro *et al*., 2016; Valiente *et al*., 2014; Wang *et al*., 2016), tyrosine kinase receptor‐independent mechanisms of Dov also occur. This includes Dov‐mediated activation of protein tyrosine phosphatase SHP‐1 and subsequent dephosphorylation of phospho‐(p)STAT3^TYR705^, resulting in the downregulation of antiapoptotic STAT3 target genes Mcl1 and survivin, and G_1_/S cell cycle promoting cyclin D1 (Chen *et al*., 2012; Tai *et al*., 2012). The inhibition of pSTAT3^Tyr705^ was shown to be dependent on SHP‐1 in colorectal (Fan *et al*., 2015) and hepatocellular carcinoma (Huang *et al*., 2016).

High‐mobility group protein A2 (HMGA2) is a nuclear nonhistone chromatin binding protein expressed in embryonic, fetal, and many cancer cells/ tissues, but is usually undetectable in normal adult somatic cells (Gattas *et al*., 1999). Its three AT‐hook DNA binding domains interact with the minor groove at AT‐rich DNA sites and have intrinsic AP/dRP lyase activities that remove cytotoxic deoxyribosephosphate (dRP) sites to facilitate expedient base excision repair (BER) and protect HMGA2^+^ embryonic stem (ES) cells and cancer (stem) cells from genomic instability and apoptosis (Natarajan *et al*., 2013; Summer *et al*., 2009). HMGA2 affects mesenchymal differentiation and ES cell proliferation (Li *et al*., 2007) and tissue‐specific overexpression of full‐length HMGA2 causes mesenchymal tumors (Mayr *et al*., 2007; Zaidi *et al*., 2006). High cellular HMGA2 levels are linked to increased malignancy, enhanced metastatic potential, and poor clinical outcome in different cancer types (Fusco and Fedele, 2007; Rogalla *et al*., 1997). Ubiquitous expression of a truncated HMGA2 mRNA lacking the 3′ untranslated region (UTR) which contains Let‐7 microRNA binding sites can result in lipomas and cancer (Battista *et al*., 1999; Yu *et al*., 2007). HMGA2 is part of a larger STAT3/LIN28/Let‐7/HMGA2 axis with important oncogenic functions in a subset of GB and breast cancer cells (Guo *et al*., 2013; Han *et al*., 2013; Mao *et al*., 2013). TCGA data analysis revealed increased gene expression of HMGA2 in the mesenchymal GB subtype compared to the glioma CpG island methylator phenotype (G‐CIMP subtype) and up to 2% of GB patients harbor HMGA2 gene amplifications as determined by cBioPortal (Jiang *et al*., 2016). Like HMGA2, the stem cell factor and Let‐7 binding protein LIN28A confers a poor prognosis in a subset of patients with GB. LIN28A‐positive GBs express high levels of HMGA2 with associated high invasiveness (Mao *et al*., 2013). HMGA2 is an important prognostic marker in patients with GB, and HMGA2 expression in GB tissues correlates with significantly reduced progression‐free survival time (Liu *et al*., 2014; Mao *et al*., 2013).

In the present study, we provide first evidence in human GB cell lines and patient GB cells that Dov attenuated the STAT3/LIN28/Let‐7/HMGA2 axis and downregulates BER factors and MGMT, a known predictor of TMZ resistance in GB. Dual or sequential treatment with Dov and TMZ enhanced the efficacy of TMZ, reduced GB cell survival, and resulted in reduced tumor sphere formation with decreased cell viability of sphere‐forming GB cells.

## 2 Materials and methods

### 2.1 Cell culture

Authenticated human glioma cell lines U251 and U87 were cultured in Dulbecco's modified Eagle's medium and F‐12 1 : 1 (DME‐F12; Hyclone, Thermo Fisher Scientific, Burlington, ON, Canada) plus 10% fetal bovine serum (FBS; Sigma, Oakville, ON, Canada). Patient GB cells were isolated from tissues obtained from GB patients treated at the Health Science Centre (Glogowska *et al*., 2013). The study was approved by the University of Manitoba and Pathology ethics boards, and written consent was obtained from the patients. Tumor sphere formation was performed and the mouse GB‐initiating cell line NF53 was established as described (Friedmann‐Morvinski *et al*., 2012; Thanasupawat *et al*., 2015).

### 2.2 Orthotopic transplantation and xenograft model

All procedures performed were approved by the IACUC. A total of 3 × 10^5^ mouse GB‐initiating NF53 cells suspended in 1–1.5 μL of HBSS were stereotactically injected into the right hippocampus of B6 mice (Friedmann‐Morvinski *et al*., 2012). Forty‐μm‐thick coronal sections from perfusion‐fixed NF53 xenografts were cut on a sliding microtome and imaged by a Zeiss LSM 710 laser scanning confocal microscope (Zeiss, Jena, Germany). Mouse tumor‐bearing brain sections perfused with 4% PFA were washed in PBS, then blocked for 1 h with 3% donkey serum, 0.25% Triton X‐100, and probed for HMGA2 (1 : 250). The intracranial U87^luc^ xenograft experiments were approved by the University of Manitoba animal ethics board and carried out as described (Thanasupawat *et al*., 2015). Brains were fixed in buffered formalin and processed for histology and immunohistochemistry (IHC).

### 2.3 Quantitative Real‐time PCR

Using 1 μg of total RNA and random primer (Promega, Madison, WI, USA), cDNA was synthesized at 65 °C for 5 min, 25 °C for 10 min, 42 °C for 50 min, and 70 °C for 15 min. Quantitative real‐time PCR (qPCR) was carried out with QuantiStudio^®^ 3 (Applied Biosystems, Thermo Fisher Scientific) using PowerUp TM SYBR^®^ green Master mix (Applied Biosystems). The following primers were used for amplification: F‐HMGA2 5′‐GCGCCTCAGAAGAGAGGAC‐3′; R‐HMGA2 5′‐TTGAGCTGCTTTAGAGGGACTC‐3′; F‐GAPDH 5′‐GTCTCCTCTGACTTCAACAGCG‐3′; R‐GAPDH 5′‐ACCACCCTGTTGCTGTAGCCAA‐3′. The relative HMGA2 expression was analyzed by comparative cycle threshold (Ct) method and normalized to GAPDH.

### 2.4 Western blot

Protein lysates were separated by SDS/PAGE and blotted onto a nitrocellulose membrane. Membranes were blocked for 1 h at room temperature (RT) with 5% milk in Tris‐buffered saline containing 0.1% Tween‐20 (TBST), pH 7.6, prior to probing with antibodies. Primary antibodies to HMGA2 (1 : 1000), HMGA1 (1 : 1000), γH2AX (1 : 1000), phospho‐STAT3 (pSTAT3^Tyr705^; 1 : 1000), total STAT3 (1 : 2000), PARP1 (1 : 2000), XRCC1 (1 : 1000), PKCα (1 : 1000), p21 (1 : 1000), α‐tubulin, MGMT (1 : 1000; all rabbit polyclonal IgG, Cell Signaling, via New England Biolabs, Whitby, ON, Canada), lamin a/c 1 : 500 (goat polyclonal antibody) and p53 (1 : 2000; both from SantaCruz, CA, USA), FEN‐1 (1 : 1000, Bethyl Laboratories, Montgomery, TX, USA), MPG (1 : 3000), Ape‐1 (1 : 2000, both Abcam, Toronto, Canada), cathepsin B (1 : 100; gift from E. Weber, Germany), LIN28A (1 : 1000; rabbit polyclonal antibody; Cedarlane, Burlington, ON, Canada), and β‐actin (1 : 10,000; Sigma) were probed overnight at 4 °C followed by incubation for 1 h at RT with HRP‐conjugated anti‐rabbit (Cell Signaling), anti‐goat, and anti‐mouse (Sigma) secondary antibodies.

### 2.5 DNA damage detection

For immunofluorescence of nuclear phosphorylated γH2AX, GB cells were fixed in 3.7% formaldehyde, permeabilized with 0.2% Triton X‐100, and nonspecific sites were blocked with 4x SSC/ 4% BSA for 1 h at RT. Primary antibodies to phosphorylated γH2AX (1 : 5000; mouse monoclonal; EMD Millipore, Mississauga, ON, Canada) were incubated with blocking buffer for 2 h at RT followed by incubation with Alexa Fluor‐594‐conjugated goat anti‐mouse IgG (Life Technologies, via Thermo Fisher Scientific) for 1 h at RT. Cells were counterstained with 4′, 6‐diamidino‐2‐phenylindole (DAPI; Sigma) and mounted with Fluoromount aqueous mounting medium. Images were acquired using a Zeiss upright fluorescence Z2 microscope (Plan‐APOCHROMAT 63x/1.4 Oil DIC objective) with monochrome CCD camera (Zeiss). For the alkaline comet assays, 50,000 U251 and U251‐HMGA2 cells were plated in six‐well plates for comet assay. Cells were incubated with different concentrations of TMZ (0.5 mm, 1 mm, 1.5 mm) for 24 hr or with low‐dose TMZ (100 μm) and Dov (2 μm) and siHMGA2 as indicated for 72 h. Cells were processed using the comet assay kit (Trevigen, Gaithersburg, MD, USA) according to the manufacturer's instructions and stained with SYBR green II (Thermo Fisher Scientific, Waltham, MA USA). Comet images were acquired using a Z2 microscope (Zeiss), and comets were analyzed using the Comet Assay IV software (Perceptive Instruments, Bury St Edmunds, UK). 50 cells per experiment and treatment were analyzed.

### 2.6 Immunohistochemistry

Forty‐μm‐thick coronal sections from perfusion‐fixed NF53 xenografts were cut using a Vibratome and processed for standard immunohistochemical staining. Mouse tumor‐bearing brain sections perfused with 4% PFA were washed in PBS, then blocked for 1 h with 3% donkey serum, 0.25% Triton X‐100, and probed for HMGA2 (1 : 250). Images were acquired with a Zeiss LSM 710 laser scanning confocal microscope (Zeiss). IHC for HMGA2 in U87 xenograft sections and human GB tissues (5 μm) was performed as described (Thanasupawat *et al*., 2015). Tissue sections were counterstained with hematoxylin prior to imaging with a Zeiss A2 microscope (Zeiss).

### 2.7 Cytotoxicity assay

For cytotoxicity assays, human GB cells and mouse NF53 cells (5000 cells per well) were seeded in a 96‐well plate, cultured overnight, and treated with HMGA2 siRNA (#SASI‐HS01 000 98053, Sigma) for 24 h, followed by 72‐h treatments with 100 μm TMZ, which represents a dose equivalent to concentrations achieved in patient GB (Portnow *et al*., 2009) and is commonly used in cell culture experiments (Kitange *et al*., 2009). For short‐term readouts of toxicity, we used 24‐h treatments with IC_50_ TMZ in human and mouse GB cells. Human GB cells were also treated with Dov (Sigma) at 1–5 μm as indicated. WST‐1 reagent (Roche Diagnostics, Laval, QC, Canada) was added and absorbance was measured at 450 nm after 4 h using a plate reader (Molecular Devices, Sunnyvale, CA, USA). For cytotoxicity in sphere‐forming cells, U87 cells were grown as tumor spheres in stem cell medium for 7 days. Spheres were dissociated as single cells and 5000 sphere‐forming cells/well in stem cell medium were seeded in a 96‐well plate for WST cytotoxicity and caspase 3/7 apoptosis assays following treatments with Dov and siHMGA2 as indicated.

The xCelligence system (ACEA Biosciences, Inc., San Diego, CA, USA) was used to determine real‐time cellular response of primary GB cells upon treatment with TMZ (100 μm) and Dov (1, 2, 5 μm) over 96 h. The system measured the cellular impedance across microelectrodes integrated on the bottom of 16‐well E‐plates. Cell viability, cell number, cell morphology, and degree of adhesion affect electrode impedance. Cells (5000 cells per well) were seeded in 96 well E‐plates containing DME/F12 media with 10% FBS. Cells were exposed to 100 μm TMZ and different concentrations of Dov (1, 2, 5 μm). Impedance measurements for real‐time monitoring occurred every 1 min.

### 2.8 Apoptosis detection

For the quantification of caspase 3/7 activity, cells (5000 cells per well) were seeded in white‐bottomed 96‐well plates and cultured overnight. Following siRNA and drug treatments for the indicated time points, caspase activation was determined with the Caspase‐Glo‐3/7 reagent (Promega) and luminescence was determined with a plate reader after 1‐h incubation at RT. We used a modified Nicoletti method to measure apoptosis by flow cytometry (Ghavami *et al*., 2009, 2010). Briefly, primary GB and U251 cells were grown in 12‐well plates (40–50%) and treated for 48 h with TMZ (100 μm), Dov (2 and 5 μm), and TMZ/Dov combination as indicated. Cells were detached by EDTA buffer (KCl: 200 mg; NaCl: 3400 mg; NaHCO3: 1100 mg; NaH_2_PO_4_. H_2_O: 70 mg; d‐glucose: 500 mg; EDTA disodium: 186 mg in 500 mL; pH = 7.4) and harvested by centrifugation at 1500 ***g***, 4 °C, for 5 min. The cells were washed once with PBS and resuspended in a hypotonic PI lysis buffer (1% sodium citrate, 0.1% Triton X‐100, 0.5 mg·mL^−1^ RNase A, 40 μg·mL^−1^ propidium iodide). Following 30‐min incubation at 30 °C, analysis was performed by flow cytometry (two‐laser FacsCalibur from BD). Cells in the sub‐G1 region containing hypodiploid DNA were considered apoptotic.

### 2.9 RNA silencing

The siRNA‐mediated silencing was performed using siLentFect lipid reagent (Bio‐Rad, Mississauga, ON, Canada) according to the manufacturer's instructions. After 72 h of HMGA2 siRNA (Sigma and Dharmacon via Thermo Fisher Scientific), MGMT siRNA (Santa Cruz), and scrambled siRNA (Cell Signaling Technologies) treatments, WST assays and western blots were performed.

### 2.10 Sphere formation assay

Glioblastoma tumor spheres were grown for 7 days in low attachment plates and then dissociated, and individual sphere‐forming cells (1,000/well) were seeded in low attachment 24‐well plates for treatments. Dov or HMGA2 siRNA treatments were performed for 3 days, and sphere formation was determined after a total of 6 days. Numbers of spheres with a diameter > 50 μm per well were counted (Hong *et al*., 2012) using a 20× objective with a phase‐contrast inverted microscope (Zeiss).

### 2.11 Colony formation assay

Two thousand cells per well were seeded in a six‐well plate in triplicate and five cycles of alternating treatments of 5 μm Dov and 100 μm TMZ were performed every 3 days followed by recovery in normal growth medium for 9 days. At the end of the treatment cycles, live cells were counted by trypan blue exclusion staining and 100 trypan blue‐negative and presumed living cells were seeded in a six‐well plate for the recovery experiment. Simultaneously control experiments were conducted in a six‐well plate in triplicate where five cycles of alternating treatment with normal growth medium and 100 μm TMZ were performed every 3 days followed by recovery in normal growth medium for 9 days. At the end of the recovery period, the number of colonies formed was counted with a 20× objective under phase contrast with an inverted microscope (Zeiss).

### 2.12 Statistical analysis

Two‐tailed unpaired t‐tests and analysis of variance (ANOVA) were performed to determine the statistical significance among the different treatment groups. Post hoc tests were performed for multiple comparisons to ensure statistical significance. A *P*‐value of less than 0.05 was considered statistically significant. Error bars represent the standard error of the mean (SEM).

## 3 Results

### 3.1 Dovitinib downregulates Lin28 and HMGA2

**Figure 1:**
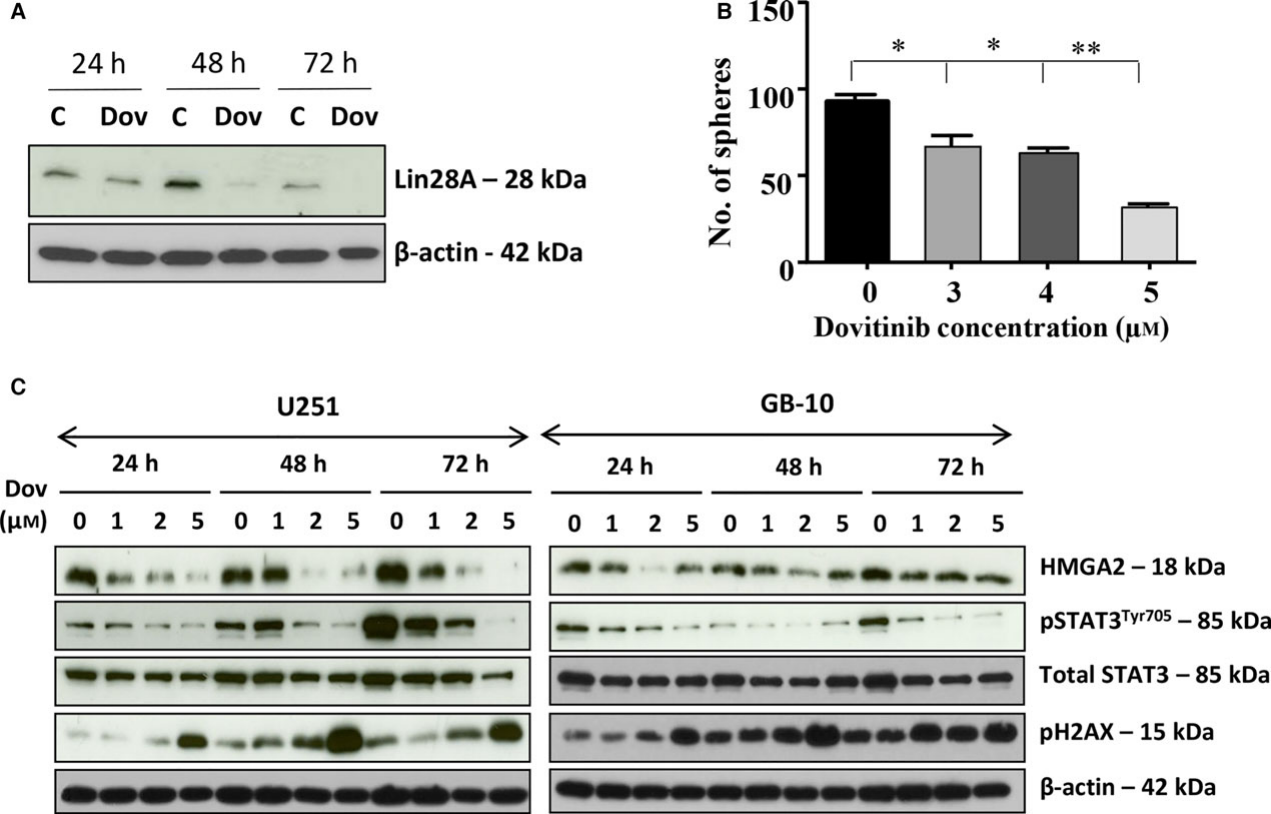
Dovitinib reduced STAT3, Lin28, and HMGA2. (A) Western blot analysis showed the downregulation of Let‐7 binding protein LIN28A upon Dov treatment (5 μm) at 48 h and 72 h in U251. (B) The number of tumor spheres with ≥50 μm diameter progressively declined with increasing Dov concentrations (3–5 μm) to reach less than 50% at 5 μm Dov, shown here for U87MG cells. (C) Dov concentrations at 1, 2, and 5 μm for 24 h, 48 h, and 72 h revealed a selective downregulation of the stem cell factor HMGA2 and pSTAT3 at Tyr705 and an increase in γH2AX shown here for U251 and GB‐10 cells. Total STAT3 remained unchanged and β‐actin served as loading control. **P < 0.01; *P < 0.05.

Although Dov has been used in clinical trials for the treatment of recurrent GB, the molecular mechanisms triggered by Dov in GB are largely unknown. We used patient GB cells and GB cell lines to show that Dov treatment downregulated both LIN28A (Fig. 1A) and HMGA2 (Fig. 1C). It was previously shown that Lin28 located upstream of HMGA2 can upregulate HMGA2 (Mao *et al*., 2013) and that HMGA2 promotes GB stem cell renewal and tumor initiation in GB cells (Zhong *et al*., 2016). In concordance with the Dov‐mediated reduction in cellular Lin28 and HMGA2 levels, we showed that Dov reduced the number of GB spheres in a concentration‐dependent manner (Fig. 1B). Similarly, we observed a reduced number of GB spheres upon siRNA‐mediated silencing of HMGA2 in GB cells (Fig. S1).

We aimed to confirm the expression of HMGA2 in patient GB cells and in human and mouse GB tissues. Brain tumor allograft sections derived from mouse GFP^+^ green fluorescent NF53 brain tumor‐initiating cells showed expression of HMGA2 (Fig. S2A) and stem cell marker nestin (Fig. S2B) in GFP^+^ NF53 brain tumor cells. IHC analysis of mouse allografts (Fig. S2C), xenografts derived from human U87MG glioma cells (Fig. S2D), and human GB tissues (Fig. S2 E**)** all showed nuclear HMGA2 expression exclusively in GB cells. Patient GB cells isolated from GB tumors and the human glioma cell lines U251 and U87MG expressed HMGA2 transcripts (Fig. S2F), and we confirmed the nuclear localization of HMGA2 protein in GB cells by western blot analysis (Fig. S2G).

Western blot analysis confirmed that Dov treatment at concentrations as low as 2 μm downregulated HMGA2 protein in GB cells (Fig. 1C). In addition to the reduced Lin28A levels, we also observed a dramatic reduction in STAT3 phosphorylation at Tyr705 under low Dov concentrations. STAT3^Tyr705^ is a known upstream regulator for the Lin28/Let7/HMGA2 oncogenic pathway (Guo *et al*., 2013). We have identified Dov as a negative regulator of this pathway in GB cells. The Dov‐mediated reduction in the STAT3/LIN28/Let‐7/HMGA2 axis coincided with an increase in cellular levels of phosphorylated γH2AX, a known marker for double‐strand (ds)DNA damage (Fig. 1C).

### 3.2 Dovitinib reduces BER factors and MGMT in GB cells

**Figure 2:**
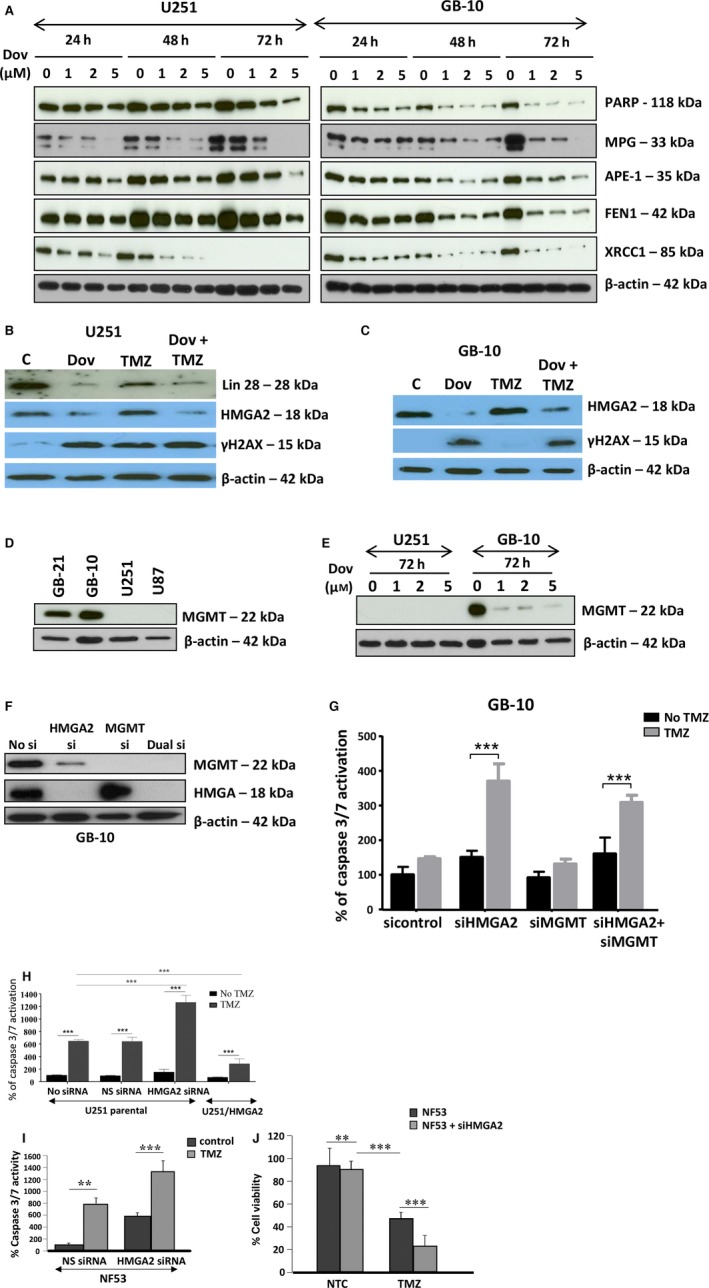
Dovitinib downregulates proteins involved in TMZ‐induced DNA damage repair. (A) Low Dov concentrations at 1, 2, and 5 μm for 24 h, 48 h, and 72 h reduced the BER proteins APE1, MPG, FEN1, PARP1, and XRCC1 with most pronounced effects seen at 72 h as shown here for U251 and GB‐10. (B) In U251, Dov at 5 μm downregulated LIN28 and HMGA2 and increased γH2AX levels at 72 h. TMZ (100 μm) alone did not downregulate Lin28 and HMGA2, but did not prevent Dov (5 μm)‐induced reduction in these proteins in U251 cells. (C) Like with U251 cells, Dov at 5 μm downregulated HMGA2 and increased γH2AX levels at 72 h. TMZ (100 μm) at 72‐h exposure did not decrease HMGA2 protein levels in GB‐10 cells and did not prevent DOV (5 μm)‐induced HMGA2 downregulation. (D) In contrast to the GB cell lines U87 and U251 which were negative for MGMT expression, patient GB cells expressed MGMT protein. (E) Dov consistently downregulated MGMT, shown here for GB‐10. (F) Western blot showing specific KD of HMGA2 or MGMT and dual silencing in GB‐10 cells. (G) Caspase 3/7 assays were performed. Individual silencing of HMGA2 and MGMT and dual HMGA2/MGMT KD showed that apoptosis was strongly induced at 24 h by TMZ (1.5 mm) in patient GB cells with silenced HMGA2, but not upon MGMT silencing or TMZ alone. (H) Compared to U251 cells with endogenous HMGA2, siHMGA2 KD significantly increased caspase 3/7 activity following treatment with TMZ. In contrast, high exogenous cellular HMGA2 levels reduced this caspase 3/7 activity upon TMZ. Similar to human GB cells, HMGA2 KD in mouse NF53 GB cells caused (I) an increase in caspase 3/7 activation, which was dramatically upregulated with TMZ, and (J) a reduction in cell viability over 24h, which was aggravated by TMZ at EC_50_ (2 mm) for 24h. Graphs show SEM from three independent experiments; ***P < 0.001; **P < 0.01.

HMGA2 has a new member of the base excision repair (BER) family of proteins (Summer *et al*., 2009). Thus, we investigated whether Dov treatment affected other BER factors in GB cells. Low‐dose Dov (1, 2, 5 μm) treatments over 72 h downregulated HMGA2 protein and decreased the BER members 3‐methyladenine DNA glycosylase (MPG), apurinic/apyrimidinic (AP) endonuclease 1 (APE‐1), flap structure‐specific endonuclease 1 (FEN1), X‐ray repair cross‐complementing 1 (XRCC1), and poly (ADP‐ribose) polymerase 1 (PARP1) (Fig. 2A). The Dov‐mediated reduction in HMGA2 and BER protein levels in GB cells is consistent and selective for LIN28A, HMGA2, and these BER factors but did not extend to other proteins previously shown to be expressed and regulated in GB cells, including PKC alpha and cathepsin B (Glogowska *et al*., 2013) (Fig. S3A). Also, we observed only a marginal reduction in protein levels for the structural relative HMGA1 (Fig. S3A). As expected, Dov treatment failed to alter HMGA2 protein levels in stable U251‐HMGA2 transfectants with constitutive exogenous overexpression of HMGA2 (Fig. S3B).

Dov exposure (5 μm) elevated levels of γH2AX, indicating increased dsDNA damage in the absence of the alkylating drug TMZ (Fig. 2B,C). TMZ also increased γH2AX levels in U251 cells (Fig. 2B), but failed to do so in patient GB cells (Fig. 2C). Patient‐derived GB‐1 and GB‐10 cells expressed MGMT protein, whereas U251 and U87MG cell lines lacked MGMT (Fig. 2D).

The clinical use of DNA‐alkylating TMZ in GB prompted us to investigate whether Dov, in addition to its role in downregulating BER factors, can also affect cellular O6‐methylguanine‐DNA methyltransferase (MGMT) levels. MGMT repairs TMZ‐induced O6‐methylguanine base modification and promotes TMZ resistance in GB (Fukushima *et al*., 2009; Hegi *et al*., 2008; Stupp *et al*., 2009). Intriguingly, Dov (1, 2, 5 μm) treatment downregulated MGMT protein in patient GB cells (Fig. 2E). This MGMT downregulation by Dov occurred in the absence of TMZ, which is known to deplete cellular MGMT protein levels in GB (Hegi *et al*., 2008) (Fig. S4A). Treatment with TMZ (100 μm) failed to alter cellular LIN28A, HMGA2, MPG, and APE‐1 levels and did not prevent the Dov‐mediated reduction in these protein levels in GB (Figs 2B,C and S4B).

We addressed whether the Dov‐mediated concurrent downregulation of HMGA2 and MGMT proteins could affect TMZ efficacy in GB cells by applying separate and combined siRNA‐mediated KD of HMGA2 and MGMT. We determined the success of this KD treatment by western blot (Fig. 2F). Neither single nor double siRNA‐mediated KD reduced viability in patient GB cells (Fig. S5). Following 24‐h exposure to TMZ, we found that single HMGA2 KD induced similar levels of caspase 3/7 activation when compared to combined KD of both HMGA2 and MGMT (Fig. 2G), demonstrating that the loss of HMGA2, not a lack of MGMT, was responsible for promoting apoptosis under TMZ. The increase in TMZ‐induced DNA damage under HMGA2 KD coincided with increased apoptosis as determined by caspase 3/7 activation (Fig. 2H). Silencing of endogenous HMGA2 increased and overexpression of exogenous HMGA2 decreased this apoptotic response under TMZ (Fig. 2H). Similar to human GB, NF53 mouse GB cells became sensitized to TMZ upon HMGA2 silencing and responded with increased caspase 3/7 activation and decreased cell viability (Fig. 2I,J), confirming a role for HMGA2 in antagonizing TMZ sensitivity. Our findings demonstrated a novel role of Dov in regulating TMZ sensitivity by attenuating expression of pSTAT3/LIN28/HMGA2, BER, and MGMT in GB cells.

### 3.3 HMGA2 expression is protective against DNA damage in GB cells

**Figure 3:**
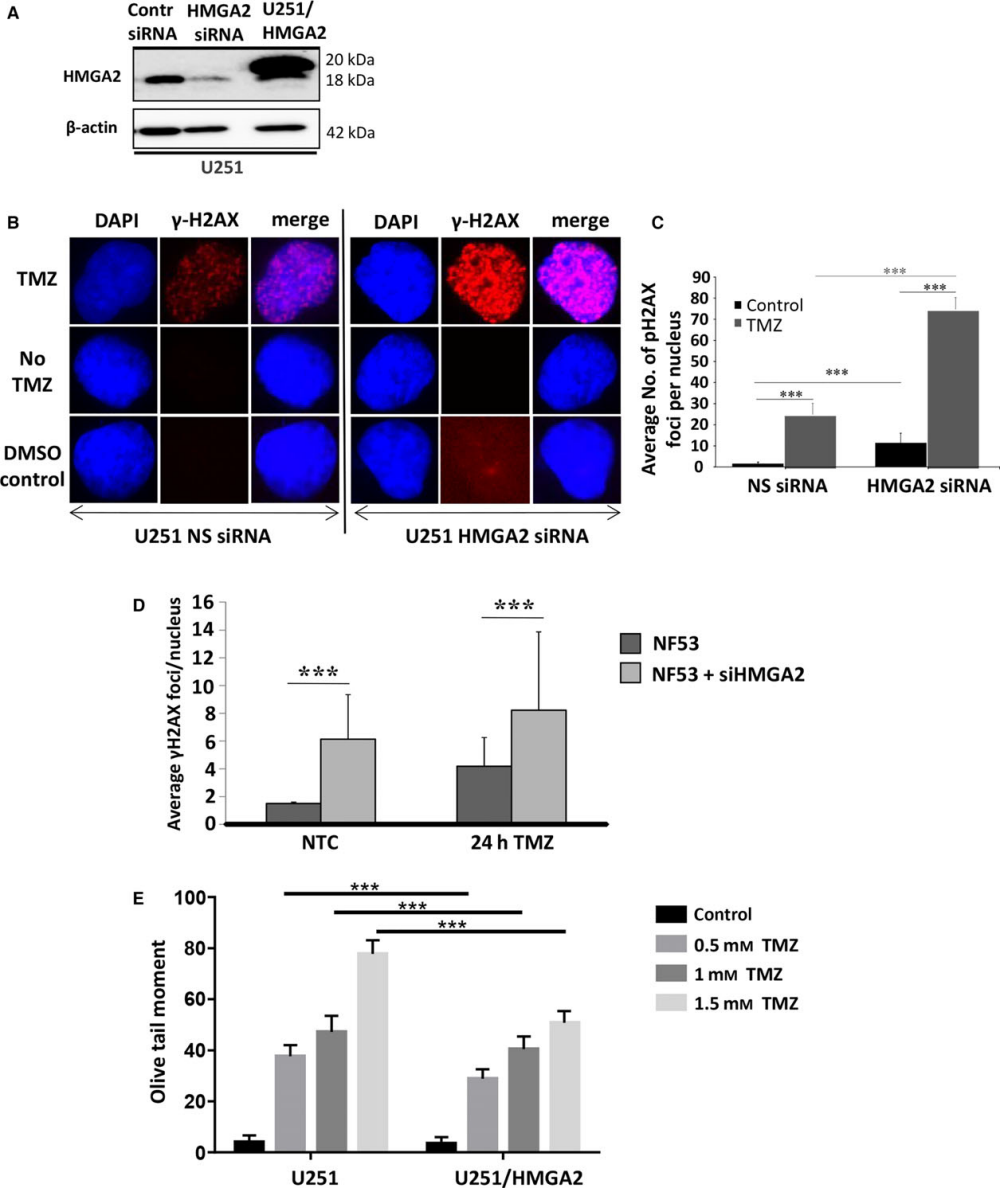
High‐mobility group protein A2 protects from TMZ‐induced DNA damage.(A) Western blot detection of successful HMGA2 KD upon specific siHMGA2 treatment in U251 cells. A scrambled sequence was used as control nonsilencing siRNA. U251 cells with stable overexpression of Flag‐tagged HMGA2 shown endogenous (18 kDa) and exogenous (20 kDa) HMGA2 were used for increased cellular HMGA2 levels. β‐Actin confirmed equal loading of protein samples. (B) TMZ treatment (1.5 mm) combined with HMGA2 KD resulted in an increased number and intensity of γ‐H2AX foci in U251 after 24h. The images show examples of single cell nuclei with γ‐H2AX foci shown in red and DAPI for nuclear counterstain in blue. (C) The average number of γ‐H2AX foci/nucleus was quantified from a total of 90 nuclei and three independent experiments (30 nuclei from each treatment and experiment). A significant increase in the average number of γ‐H2AX foci/nucleus was detected upon siHMGA2 vs. nonsilencing siRNA‐treated cells (black bars). An additional significant increase occurred upon TMZ treatment in U251 with siHMGA2 KD (gray bars). (D) As in human GB cells, HMGA2 silencing in mouse NF53 GB cells induced DNA damage as determined by increased number of nuclear γ‐H2AX foci. The DNA damage was further aggravated under TMZ at EC_50_ (2 mm) for 24h. (E) Alkaline comet assays in U251 cells demonstrated that treatment with TMZ at 0.5, 1, and 1.5 mm for 24 h significantly increased DNA strand breaks as quantified by the olive tail moment. Exogenous HMGA2 overexpression diminished the TMZ‐induced DNA damage compared to endogenous HMGA2 levels. Graphs show SEM from three independent experiments; ***P < 0.001.

Our data demonstrated that HMGA2 protects GB cells against apoptosis induction by TMZ (Fig. 2G,H). To assess whether the DNA repair function of HMGA2 contributed to this protective effect, we investigated the TMZ‐induced DNA damage in GB cells with high and low HMGA2 levels. We used U251 cells to silence endogenous HMGA2 using specific HMGA2 siRNA and confirmed by western blots the specific and efficient KD of HMGA2 within 48 h of siHMGA2 treatment (Fig. 3A). We also created U251 transfectants with stable expression of Flag‐tagged HMGA2 (Fig. 3A). To assess whether HMGA2 silencing increased TMZ‐induced DNA damage, we quantified γH2AX foci in U251 (Sharma *et al*., 2012). Upon 24 h of TMZ treatment (1.5 mm), increased numbers of bright γH2AX foci were detected in U251 (Fig. 3B). Quantification of these nuclear γH2AX foci revealed that HMGA2 KD alone caused a significant increase in the number of γH2AX foci in U251 when compared to nonsilencing control siRNA‐treated cells (Fig. 3C). We observed a further dramatic increase in γH2AX foci in HMGA2‐silenced GB cells upon TMZ treatment, indicating significant dsDNA damage in HMGA2‐depleted glioma cells (Fig. 3C). Similarly, NF53 mouse GB cells showed increased nuclear γH2AX foci upon HMGA2 KD and a further increase upon exposure to TMZ (Fig. 3D). The DNA strand breaks under TMZ exposure in U251 cells were confirmed by alkaline comet assays. We detected an increase in the olive tail moment when GB cells were exposed to increasing concentrations of TMZ (Fig. 3E). While the olive tail moment obtained with U251‐HMGA2 transfectants also increased with higher TMZ concentrations, HMGA2 overexpression significantly mitigated dsDNA strand breaks compared to U251 cells (Fig. 3E). These results revealed a new role for HMGA2 in antagonizing TMZ‐induced DNA damage in both human and mouse GB. Importantly, these results suggested that the Dov‐mediated downregulation of HMGA2 may be a promising molecular mechanism to increase GB cell sensitivity to TMZ.

### 3.4 Dovitinib sensitizes GB cells to TMZ‐induced apoptosis

**Figure 4:**
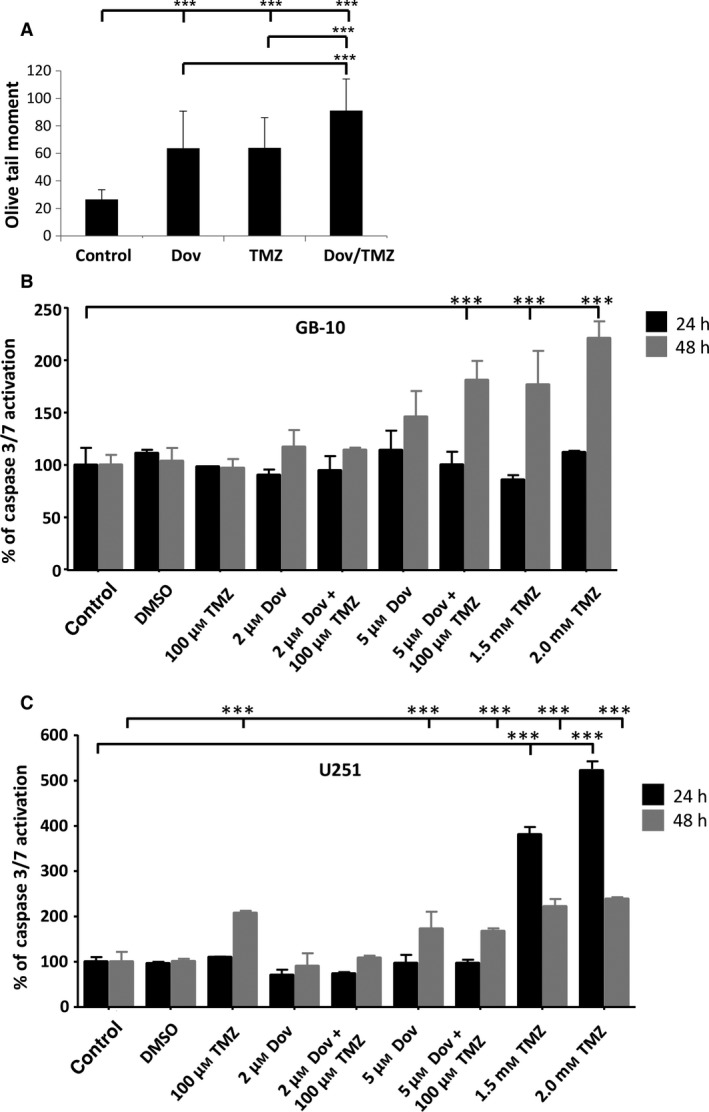
Combined TMZ and dovitinib treatment induces apoptosis. (A) Alkaline comet assays showed that treatment with Dov (2 μm) alone over 72 h, similar to treatment with TMZ alone, induced significantly increased DNA damage as quantified by the olive tail moment. The combined Dov/TMZ exposure over 72 h showed further increase in DNA damage. (B) TMZ (100 μm) failed to induce caspase 3/7 activation in GB‐10 patient cells at 24 h and 48 h, but when combined with Dov (5 μm) triggered apoptosis at 48 h. High TMZ doses (1.5 and 2 mm) were used as positive control for caspase 3/7 activation at 24h and 48 h. (C) In U251 cells, both TMZ (100 μm) and Dov (5 μm) induced caspase 3/7 activation after 48 h but combined Dov plus TMZ treatment did not result in a further detectable increase in caspase 3/7 activation. High TMZ doses (1.5 and 2 mm) were used as positive control for caspase 3/7 activation at 24h and 48 h. Graphs show SEM from three independent experiments; ***P < 0.001.

**Figure 5:**
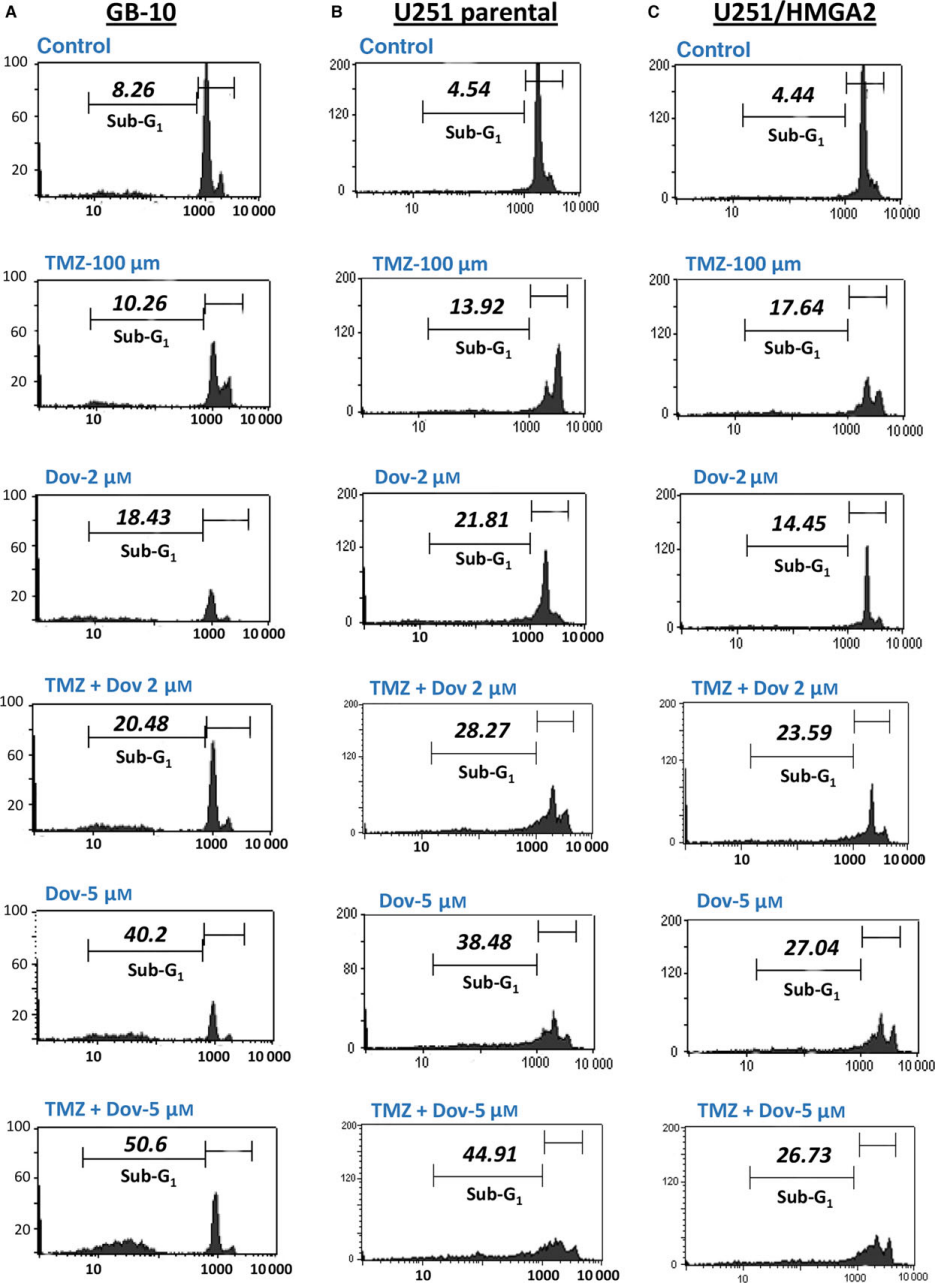
Combined TMZ and dovitinib treatment increases cell death. GB cells were treated with 100 μm TMZ alone, 2 μm and 5 μm Dov alone or in combination with 100 μm TMZ and the percentage of apoptotic cells (sub‐G1 population) was quantified by PI flow cytometry after 48 h in GB‐10 (A), U251 (B), and U251/HMGA2‐overexpressing (C) cells. The % apoptotic cell fraction gated in M2 (sub‐G1 subpopulation) is indicated for each cell line and treatment. HMGA2 overexpression mitigated the apoptotic response to Dov alone and to the combined Dov/TMZ treatment, and this effect was pronounced at 5 μm Dov (C). Representative FACS results of three independent experiments are shown.

The reduction in BER proteins and MGMT observed with Dov treatment encouraged us to investigate whether Dov treatment would increase GB cell sensitivity toward TMZ. Comet assays revealed that Dov (2 μm) and TMZ (100 μm) single treatment induced dsDNA strand breaks and dual Dov/TMZ treatment further increased this dsDNA damage upon treatment of U251 (Fig. 4A). In GB‐10 patient cells, TMZ (100 μm) treatment failed to induce caspase 3/7 activation, whereas the addition of Dov (5 μm) triggered caspase 3/7 activation at 48 h (Fig. 4B). In U251 cells, both TMZ (100 μm) and Dov (5 μm) single treatments induced caspase 3/7 activation after 48 h, but combined Dov/TMZ treatment failed to show an additive effect on caspase 3/7 activation (Fig. 4C). As caspase‐independent mechanisms may contribute to apoptosis in patient GB cells, we used flow cytometry detection to quantify the PI‐positive sub‐G1 population of apoptotic cells following 48 h of treatment. Upon dual treatment with TMZ (100 μm) and Dov (2 μm) of GB‐10, the apoptotic cell fraction in FACS analysis (20.48%) was similar to Dov monotherapy (2 μm; 18.43%), but had doubled when compared to single treatment with TMZ (100 μm; 10.26%) (Fig. 5A). When the Dov concentration was increased to 5 μm, apoptosis increased further under combined Dov/TMZ (50.6%) and Dov alone (40.2%). In U251 cells, the apoptotic cell fraction was higher than in GB 10 cells with single TMZ (100 μm; 13.92%) or DOV (2 μm; 21.81%) exposure and reached 28.27% with the combined treatment (Fig. 5B), demonstrating a strong proapoptotic effect of Dov and the combined Dov/TMZ treatment. High cellular levels of HMGA2, as tested for the U251‐HMGA2‐overexpressing clone, reduced the apoptotic cell fraction of the combined Dov/TMZ treatment (23.59%) compared to U251 parental cells (28.27%) at 2 μm Dov (Fig. 5C). This antiapoptotic effect of HMGA2 was more pronounced in U251‐HMGA2 exposed to 5 μm Dov alone (27.04%) and Dov/TMZ combined treatment (26.73%) as compared to U251 parental cells with 38.48% and 44.91%, respectively. These results suggested that a combined Dov/TMZ treatment may be advantageous in reducing GB cell survival.

### 3.5 Combined treatment with dovitinib and TMZ reduces GB cell survival

**Figure 6:**
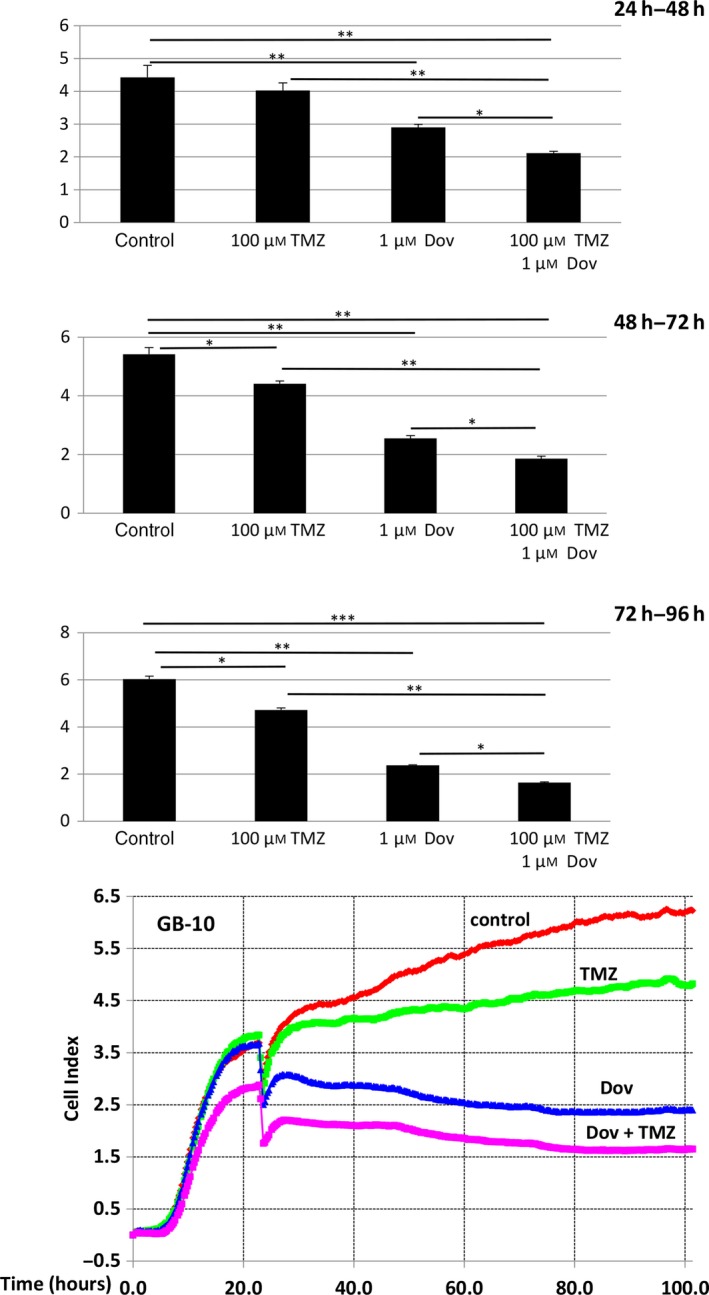
Dovitinib enhances TMZ‐induced cytotoxicity. The xCelligence impedance‐based real‐time cell monitoring over 96 h under low‐dose Dov (1 μm) and TMZ (100 μm) demonstrated that Dov plus TMZ combined had increased cytotoxic effects on GB‐10 cells and resulted in stunted cell proliferation when compared to either drug alone. TMZ (100 μm) alone only moderately blocked GB cell growth. The column graphs show the cell index (*Y*‐axis) during 24 h, 48 h, and 72 h of exposure averaged from three independent experiments. ***P < 0.001; **P < 0.01; *P < 0.05.

**Figure 7:**
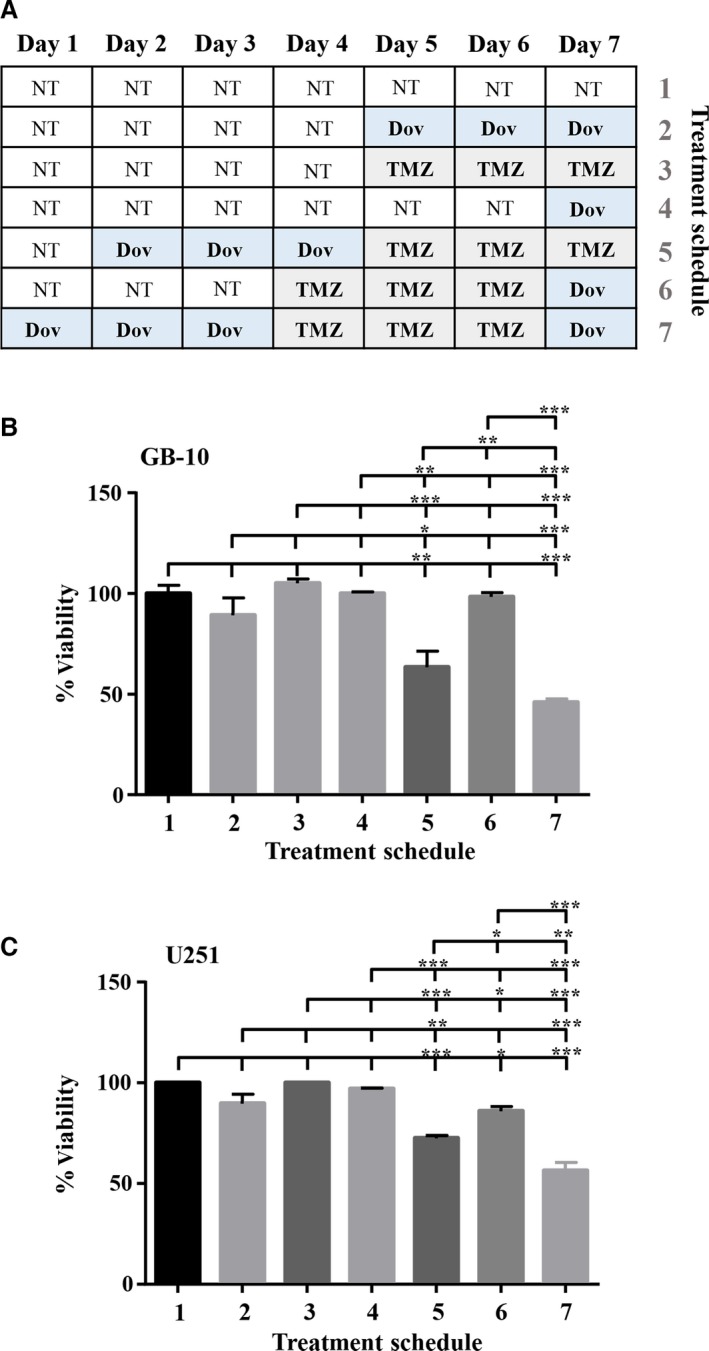
Sequential dovitinib/TMZ treatment increases toxicity. We performed sequential treatment with Dov and TMZ. **(**A**)** Schematic representation of the sequential treatment strategy over 7 days using Dov (5 μm) and TMZ (100 μm) in GB‐10 and U251. WST assays were performed to determine the percentage cell viability of (B) GB‐10 and (C) U251 after pretreatment with 5 μm Dov for 3 days, followed by 3 days with 100 μm TMZ and 1 day of 5 μm Dov. No treatment controls (NT) and single TMZ or Dov treatment controls were employed. Graphs show SEM from three independent experiments; ***P < 0.001; **P < 0.01; *P < 0.05.

Real‐time cell survival assays (RTCA) performed with patient GB cells showed that TMZ (100 μm) reduced the cell index, a measure of cell viability, over time but still allowed a steady cell proliferation over 96 h to reduce cell viability by only 23% (Fig. 6). Single Dov exposure at low concentrations (1 μm) and the combined Dov/TMZ treatment significantly reduced the cell index, resulting in a 70% reduction in cell viability over the monitored 96‐h time period (Fig. 6). We reasoned that downregulation of HMGA2, MGMT, and BER members under low‐dose Dov treatment may sensitize GB cells toward TMZ. To test this, we devised a sequential treatment starting with ‘Dov priming’ to attenuate cellular HMGA2, BER, and MGMT capacities, followed by TMZ exposure of the GB cells (Fig. 7A). Upon 72 h of pretreatment with Dov (5 μm) and subsequent 72‐h exposure to TMZ (100 μm), we observed a 40% reduction in cell viability in patient GB cells (Fig. 7B), which increased further to 50% upon subsequent additional Dov treatment (Fig. 7B). Similar results were found in U251 cells (Fig. 7C). Low‐dose TMZ treatment alone or the reversed sequence of exposure resulted in <10% loss of viability in GB cells (Fig. 7B,C).

**Figure 8:**
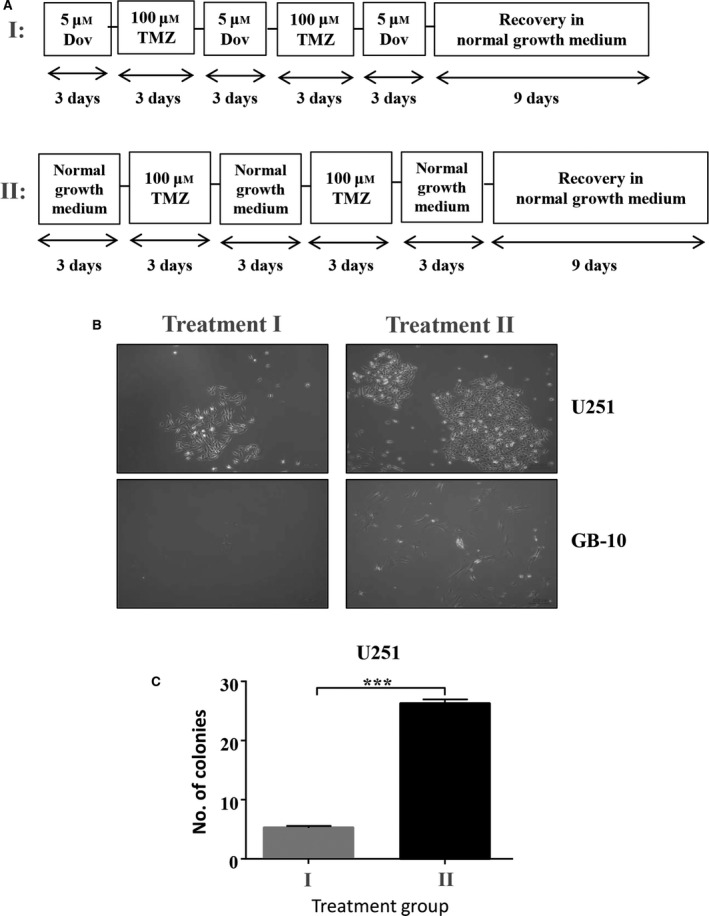
Alternating dovitinib/TMZ treatments reduce cell survival upon recovery. (A) Schematic representation of the alternating Dov (5 μm) and TMZ (100 μm) treatment plan, 3 days each and 1 day of 5 μm Dov, for U251 and GB‐10 followed by a nine‐day cell recovery phase in normal medium without drugs. Survival of GB cells was determined by their ability to form colonies as quantified at the end of the recovery time. (B) Representative phase‐contrast images are shown. (C) Alternating treatments with Dov and TMZ every 3 days for a total of 15 days followed by a recovery phase for 9 days (I) significantly reduced the number of colonies by >75% when compared to alternating treatment with TMZ alone (II), as shown for U251. Graphs show SEM from three independent experiments; ***P < 0.001.

Next, we investigated whether a sequential treatment regimen can affect long‐term survival of GB cells. To assess whether the observed loss in GB viability coincided with reduced GB cell survival after recovery from treatments, we performed colony formation assays with U251 cells and patient GB cells. We exposed GB cells to alternating low‐dose Dov followed by low‐dose TMZ treatments for 3 days each over a 15‐day time period and determined colony formation after a 9‐day recovery phase in growth medium without drugs added **(**Fig. 8A‐I). Colony formation was compared to treatment with low‐dose TMZ alone but lacking the Dov priming and intermittent Dov exposure steps prior to the recovery phase (Fig. 8A‐II). Compared to TMZ treatment alone, Dov priming with subsequent alternation of TMZ and Dov exposure drastically reduced colony formation in U251 and almost inhibited survival of patient GB‐10 cells (Fig. 8B,C). We confirmed that MGMT and HMGA2 downregulation in GB‐10 and HMGA2 downregulation in U251 persisted throughout the entire sequential alternating Dov/TMZ treatment regimen (Fig. S6). Based on these results, we concluded that this novel sequential dual‐hit Dov/TMZ treatment increases TMZ efficacy, reduces HMGA2‐mediated antiapoptotic activity, and dramatically reduces the long‐term survival in GB cells following TMZ treatment. Importantly, Dov priming was effective in MGMT+ GB cells and independent of P53 mutation status (Fig. S7). Furthermore, our novel finding that HMGA2 also mitigated DNA damage induced by radiation (Fig. S8) may suggest that Dov‐mediated downregulation of HMGA2 in GB cells may serve as a molecular mechanism to radiosensitize GB.

## 4 Discussion

The current standard treatment for GB includes surgical debulking followed by treatment with the DNA‐alkylating agent TMZ and radiation therapy. However, this therapy frequently fails to prevent the development of resistance and fatal recurrences. There is an urgent need to identify drugs which help to sensitize GB cells to chemo‐ and radiation therapy. Based on its multikinase inhibitory function and the ability to cross the BBB (Schafer *et al*., 2016), the FDA‐approved drug dovitinib (TKI258, CHIR258) has been tested as monotherapy in clinical trials for patients with advanced and recurrent GB in Germany [NCT01972750] and the United States [NCT01753713]. First results from the German trial demonstrated efficacy in some recurrent GB patients and recommended additional personalized trials (Schafer *et al*., 2016). Currently, no information is available on the molecular mechanisms by which Dov affects GB cells.

Our data in GB cells showed that Dov downregulates the stem cell factor and RNA‐binding protein Lin28A and its target HMGA2, identifying a hitherto unknown role of Dov as an inhibitor of the LIN28/Let‐7/HMGA2 axis in human GB. In agreement with the well‐known function of HMGA2 in promoting self‐renewal of stem cells (Zhong *et al*., 2016) and GB tumor initiation capability (Kaur *et al*., 2016), Dov attenuated GB sphere formation and increased apoptosis in sphere‐forming GB cells. HMGA2 expression is controlled by specific Let‐7 microRNA members that bind to the 3′UTR of HMGA2 to cause reduced mRNA stability and translation (Hammond and Sharpless, 2008). LIN28 upregulates HMGA2 by inactivating Let‐7 family members (Droge and Davey, 2008; Hammond and Sharpless, 2008; Weingart *et al*., 2015) and LIN28 is expressed in GB patients with poor prognosis (Qin *et al*., 2014). Furthermore, HMGA2 is present in a LIN28A expressing subset of GB (Mao *et al*., 2013). We confirmed the expression of HMGA2 in nestin^+^ GB progenitors in our mouse allografts and showed nuclear HMGA2 expression in human orthotopic GB xenografts and human GB tissues. Dov also reduces the cellular levels of phospho‐(p)STAT^Tyr705^ in GB cells. Thus, by altering the phosphorylation status of STAT3, Dov targets the STAT3/LIN28/Let‐7/HMGA2 axis, which is emerging as an important oncogenic pathway for HMGA2 regulation in a subset of GB and breast cancer cells (Guo *et al*., 2013; Han *et al*., 2016; Mao *et al*., 2013). Dov was shown to activate the protein tyrosine phosphatase SHP‐1, which resulted in the consecutive dephosphorylation of pSTAT3^TYR705^ and downregulation of the antiapoptotic STAT3 target genes Mcl1 and survivin, and G_1_/S cell cycle factor cyclin D1 (Chen *et al*., 2012; Tai *et al*., 2012). STAT3 activation is known to promote self‐renewal and tumorigenicity in stem‐like GB cells (Gong *et al*., 2015) and is associated with radiosensitivity in GB (Maachani *et al*., 2016). The inactivation of pSTAT3^Tyr705^ was shown to depend on SHP‐1 in colorectal (Fan *et al*., 2015) and hepatocellular carcinoma (Huang *et al*., 2016). Dov‐mediated downregulation of LIN28A and HMGA2 may, thus, be the result of enhanced SHP‐1 in GB cells. STAT3 activation is promoted by cytokines such as IL‐6 (Zhong *et al*., 1994), and the poor survival of GB patients with upregulated IL‐6 and HMGA2 (Chiou *et al*., 2013) demonstrates the urgent need for therapeutic targeting of regulatory pathways upstream of HMGA2 in patients with GB.

We showed that Dov caused increased DNA damage in GB cells as detected by γH2AX foci and comet assays. The observed inhibitory effects of Dov on BER factor, HMGA2, and MGMT protein expression indicate that the origin of the Dov‐mediated dsDNA breaks is likely complex and may involve different mechanisms. Apart from its multikinase inhibitory activity, Dov is an inhibitor of the catalytic relaxation activity of topoisomerase I and blocks the decatenation activity of topoisomerase II (Hasinoff *et al*., 2012). The resulting single‐ and double‐strand DNA breaks cause stalled DNA replication/transcription and trigger apoptosis (Nitiss, 2009). The Dov‐induced decrease in BER factors (MPG, APE‐1, FEN1, PARP1, XRCC1) is anticipated to attenuate single‐strand BER functions and enhance the occurrence of lethal dsDNA breaks (Davidson *et al*., 2013; Woodhouse *et al*., 2008). Another way of Dov to initiate DNA damage may involve HMGA2, which has recently been shown to augment topoisomerase I activity in a ternary complex with DNA and antagonize the topoisomerase I poison irinotecan/SN‐38 (Peter *et al*., 2016). Attenuated HMGA2 expression may negatively affect topoisomerase I functions in GB. It remains to be seen whether Dov can alter topoisomerase I expression in GB. Reduced HMGA2 levels under Dov are also expected to compromise the ability of HMGA2 to support BER (Summer *et al*., 2009).

Reduced BER capacity under Dov is expected to impair the repair of the three TMZ‐induced alkylated DNA base modifications N7‐methylguanine (N7‐MeG; 60–80%), N3‐methyladenine (N3‐MeA; 10–20%), and O6‐methylguanine (O6‐MeG; 5–10%) (Bobola *et al*., 2012). While the former two lesions are repaired by BER (Tang *et al*., 2011), O6‐methylguanine‐DNA methyltransferase (MGMT) specifically repairs O6‐MeG sites and is known to promote TMZ resistance in GB (Fukushima *et al*., 2009; Hegi *et al*., 2008; Stupp *et al*., 2009). Dov causes sustained downregulation of MGMT, suggesting that Dov can compromise this TMZ resistance mechanism in GB. These findings led us to hypothesize that Dov can sensitize GB toward TMZ‐induced cell death. MGMT^neg^ U251 cells were more sensitive to single TMZ or Dov treatment than MGMT+ patient GB cells as shown by caspase 3/7 activation assays. Upon TMZ treatment, MGMT^neg^ U251 glioma cells undergo mismatch repair (MMR) cycles with resulting dsDNA breaks and caspase 3/7‐dependent apoptosis in subsequent cell cycles (Quiros *et al*., 2010). Both MGMT^neg^ U251 and MGMT^+^ patient GB cells showed a significant increase in caspase activation and PI‐positive apoptotic cell fraction when exposed to dual low‐dose Dov/TMZ treatment. Targeted single and dual siRNA KD of HMGA2 and MGMT demonstrated that HMGA2, not MGMT, protected GB cells from TMZ‐induced apoptosis and this protective role of HMGA2 was independent of p53 activity status. We and others have previously demonstrated an antiapoptotic function of HMGA2 (Natarajan *et al*., 2013; Shi *et al*., 2016). Thus, the reduced HMGA2 protein levels contributed to increased apoptosis in GB cells exposed to dual Dov/TMZ treatment.

We devised a sequential treatment regimen starting with ‘Dov priming’ to attenuate DNA repair and antiapoptotic capacities, followed by TMZ treatment to induce DNA base lesions in an attempt to improve the efficacy of both drugs in GB cells. Sequential Dov/TMZ treatment significantly reduced cell viability by 40% and 30% in patient GB cells and U251 cells, respectively, compared to TMZ or Dov single treatments. Importantly, just two cycles of sequential Dov/TMZ treatment followed by a final Dov treatment and subsequent 9 days of treatment‐free recovery period significantly reduced or almost inhibited colony formation with U251 and patient GB cells, respectively. These results showed that the sequential dual‐hit Dov/TMZ treatment can dramatically reduce or inhibit long‐term survival of GB cells. This effect was independent of the MGMT status of the GB cells. The increased dsDNA damage and enhanced apoptotic cell death upon combined Dov/TMZ treatment were both linked to diminished cellular HMGA2 levels. U251‐HMGA2 transfectants with overexpression of exogenous HMGA2 were partially protected from this increased DNA damage and apoptosis upon dual Dov/TMZ treatment. Importantly, we observed a similar protective effect of HMGA2 on radiation‐induced DNA damage in GB cells. Recently, Dov has been described as a radiosensitizer for hepatocellular carcinoma (Huang *et al*., 2016). Our discovery suggests that HMGA2 may facilitate this radiosensitizing function of Dov in GB.

## 5 Conclusion

Dov treatment attenuated the STAT3/LIN28/Let‐7/HMGA2 axis and expression of specific BER factors and MGMT proteins. This resulted in increased TMZ sensitivity, reduced DNA repair capacity, increased dsDNA breaks and the induction of apoptosis in mouse and human GB cells. The combination of Dov and TMZ treatments reduced GB growth and self‐renewal capacity and severely compromised the recovery of GB cells. When administered in an alternating sequence, a regimen of ‘Dov priming’ and subsequent TMZ‐induced DNA alkylation may improve the therapeutic efficacy of current stand‐alone TMZ treatments, particularly in patients with MGMT+ GB and functional p53 status who are expected to have higher TMZ resistance. The concept of ‘Dov priming’ is a promising novel therapeutic strategy to improve TMZ efficacy in patients with GB.

## Author contributions

TK and SHK designed the project, interpreted the data, wrote the manuscript, and provided funding. TT, SN, AR, and AG acquired the data. SG performed and designed flow cytometry. HB critically read the manuscript. IMV provided the mouse model. JK, JB, and MP provided surgical GB tissues and patient information. SK made the pathology assessment of tumors.

## Supporting information


**Fig. S1.** HMGA2 silencing impairs sphere formation.

**Fig. S2.** HMGA2 is expressed in human and mouse GB cells.

**Fig. S3.** Dovitinib mediated specific down‐regulation of endogenous HMGA2.

**Fig. S4.** Temozolomide depletes MGMT but does not regulate BER factors in GB.

**Fig. S5.** HMGA2 and MGMT knockdown by siRNA are not toxic to GB cells.

**Fig. S6.** Dovitinib caused lasting MGMT protein reduction.

**Fig. S7.** P53 expression and functionality.

**Fig. S8.** HMGA2 protects against radiation‐induced DNA damage in GB cells.
