# Supplementary figures and images for: Dovitinib enhances temozolomide efficacy in glioblastoma cells
Source: Mol Oncol. 2017 Jun 5;11(8):1078–98. doi: 10.1002/1878-0261.12076 (PMC5537714; doi:10.1002/1878-0261.12076)

## Suppl. Fig. 1

**A.**

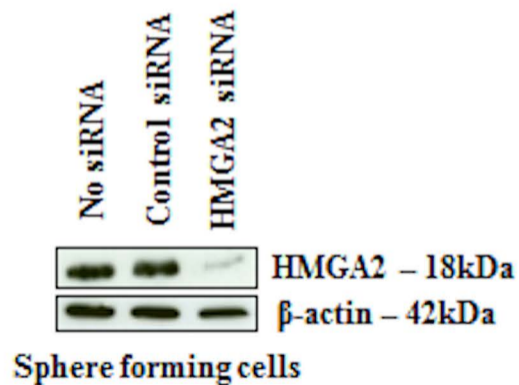

**B.**

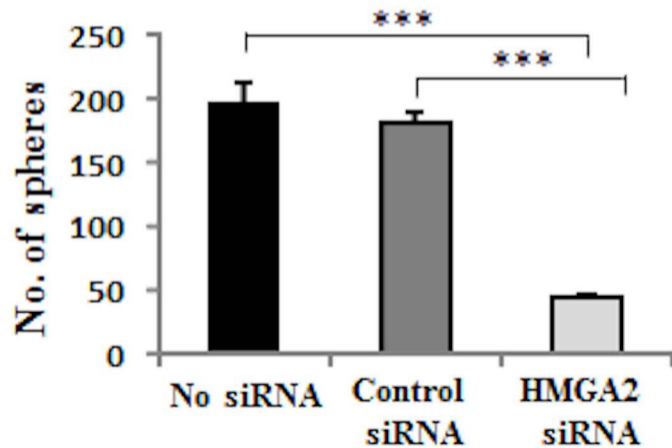

Supplement: Supplementary file 1 — Fig. S1. HMGA2 silencing impairs sphere formation. [file MOL2-11-1078-s001.pdf]

Suppl. Fig. 2

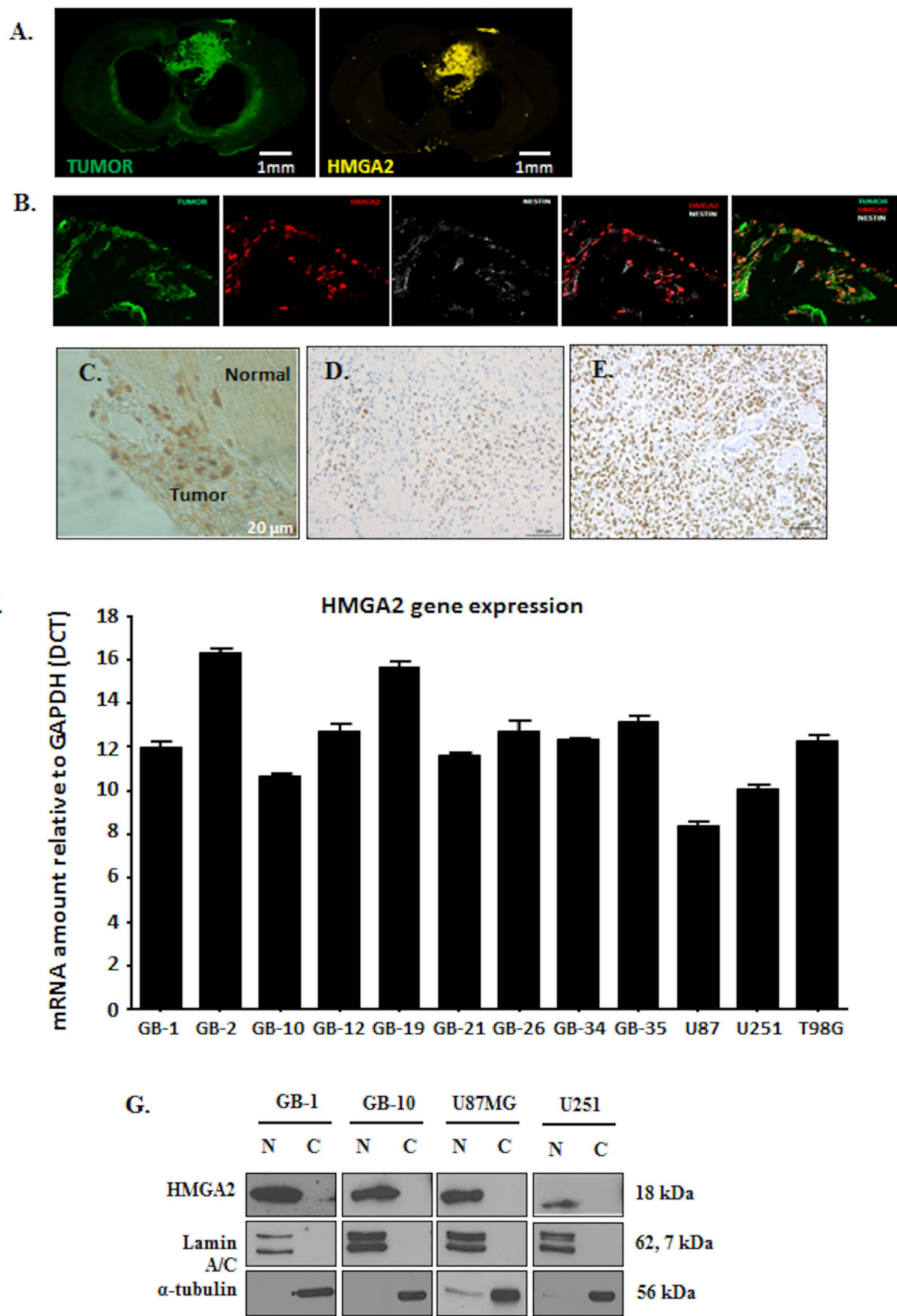

Supplement: Supplementary file 2 — Fig. S2. HMGA2 is expressed in human and mouse GB cells. [file MOL2-11-1078-s002.pdf]

# Suppl. Fig. 4

A.

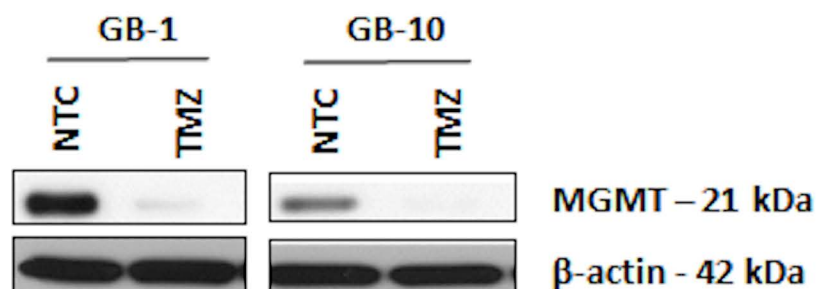

B.

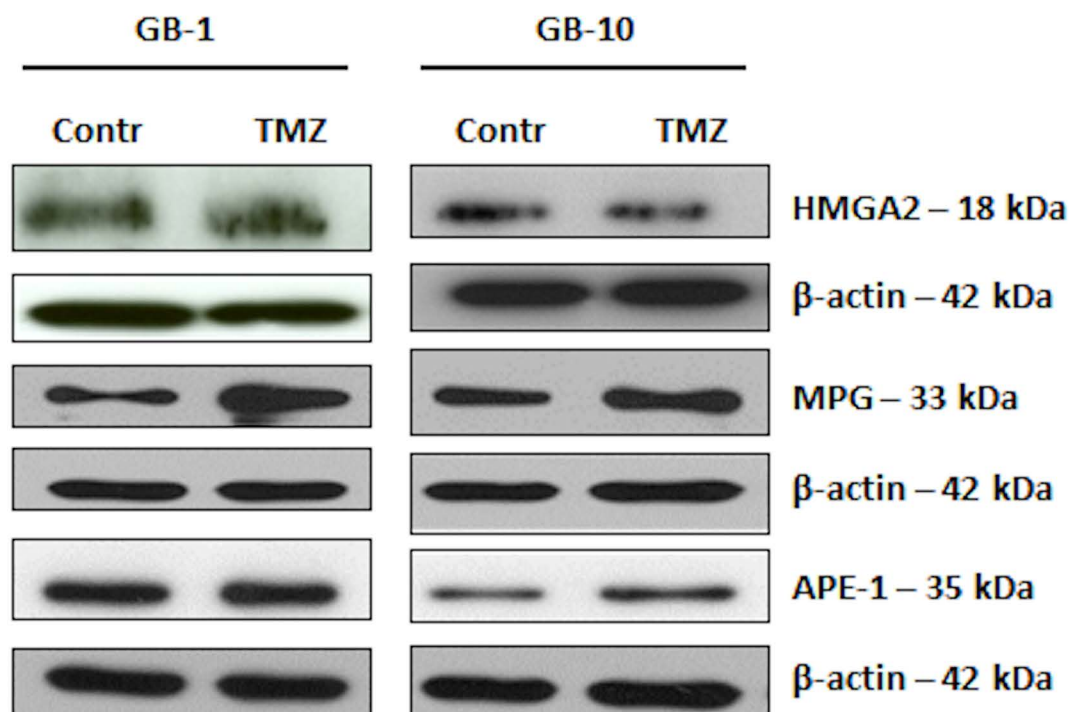

Supplement: Supplementary file 4 — Fig. S4. Temozolomide depletes MGMT but does not regulate BER factors in GB. [file MOL2-11-1078-s004.pdf]

**Suppl. Fig. 5**

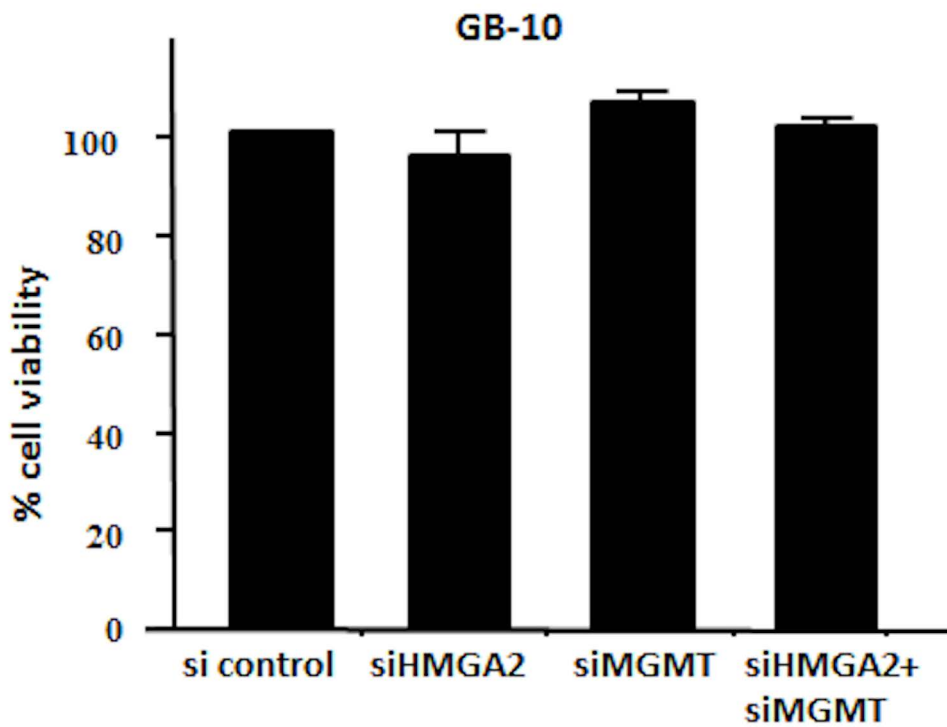

Supplement: Supplementary file 5 — Fig. S5. HMGA2 and MGMT knockdown by siRNA are not toxic to GB cells. [file MOL2-11-1078-s005.pdf]

# Suppl. Fig.6

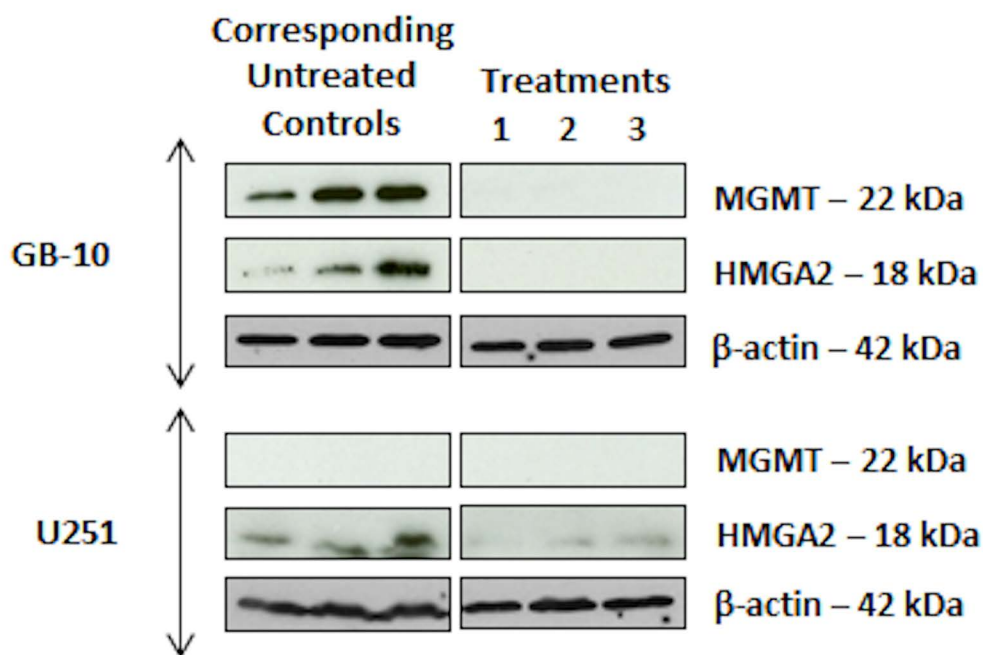

Treatments:

1: 3d Dov

2: 3d Dov + 3d TMZ

3: 3d Dov + 3d TMZ + 1d Dov

Supplement: Supplementary file 6 — Fig. S6. Dovitinib caused lasting MGMT protein reduction. [file MOL2-11-1078-s006.pdf]

**Suppl. Fig.7**

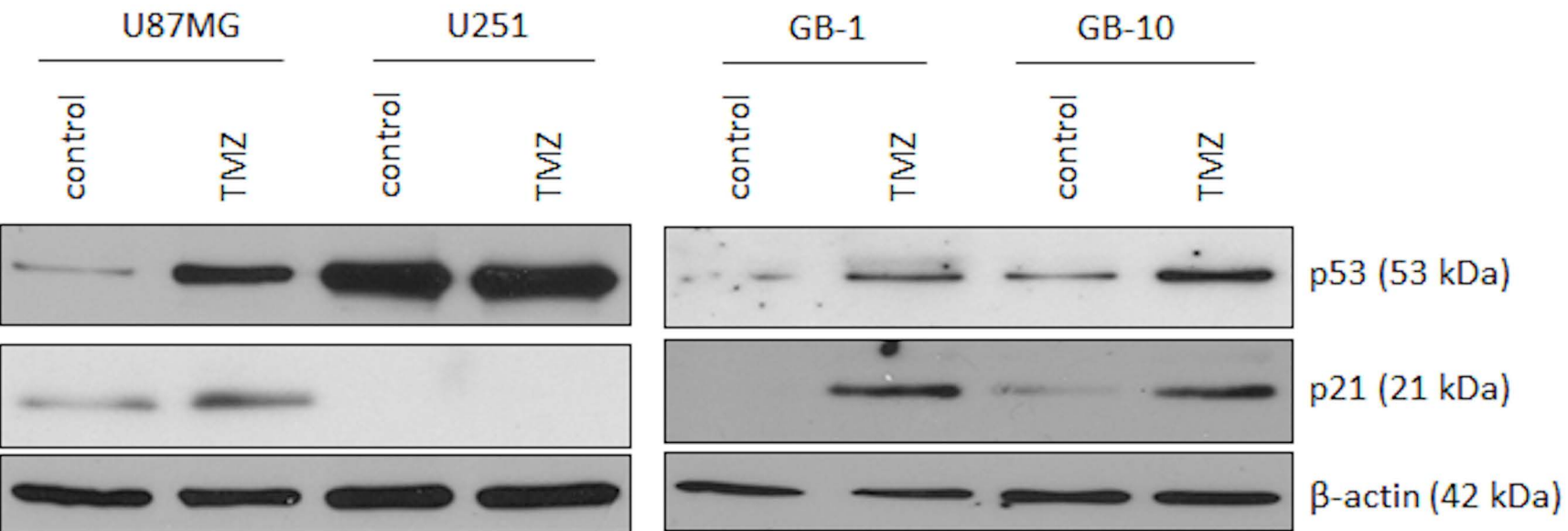

Supplement: Supplementary file 7 — Fig. S7. P53 expression and functionality. [file MOL2-11-1078-s007.pdf]

Suppl. Fig. 8

A.

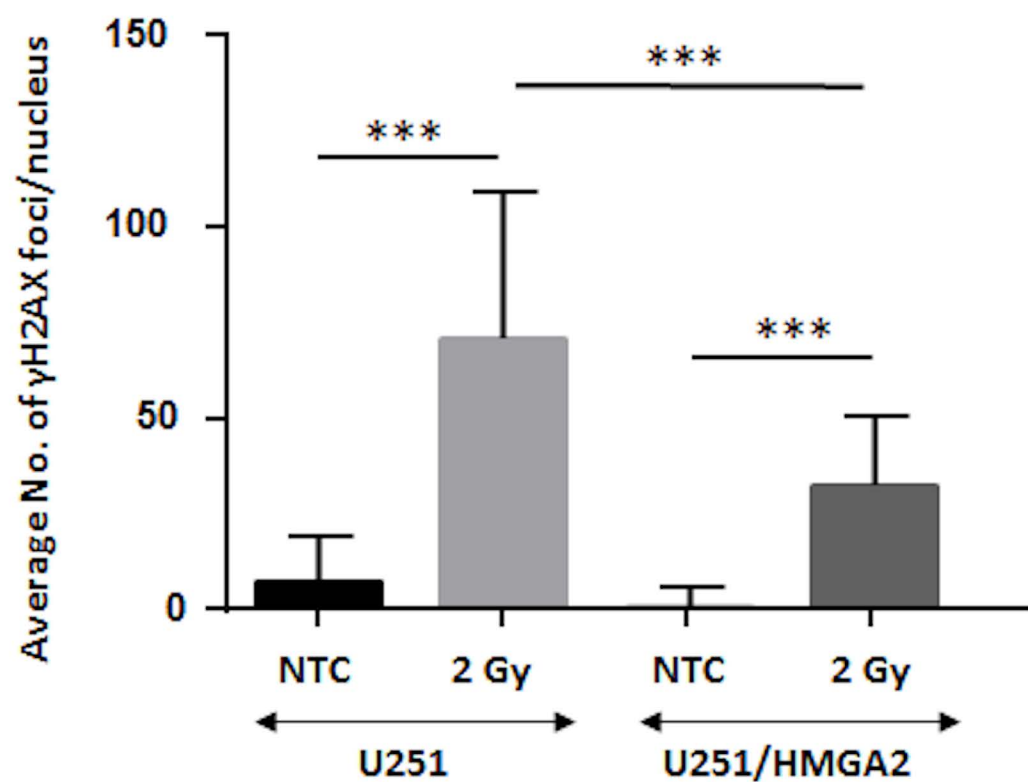

B.

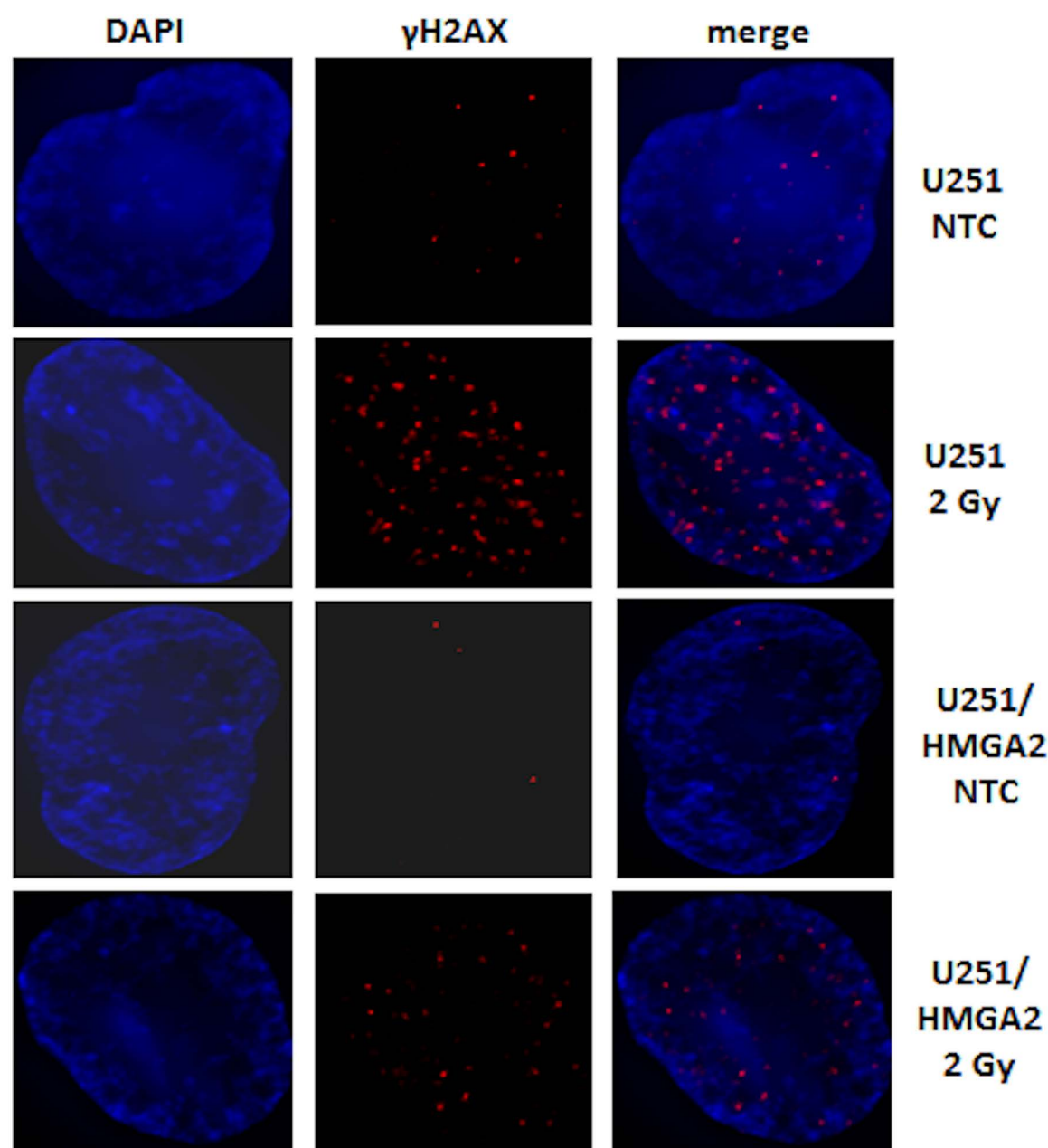

Supplement: Supplementary file 8 — Fig. S8. HMGA2 protects against radiation‐induced DNA damage in GB cells. [file MOL2-11-1078-s008.pdf]
