# Supplementary material for: Dovitinib enhances temozolomide efficacy in glioblastoma cells
Source: Mol Oncol. 2017 Jun 5;11(8):1078–98. doi: 10.1002/1878-0261.12076 (PMC5537714; doi:10.1002/1878-0261.12076)

Suppl. Fig. 3

A.

U251 Parental

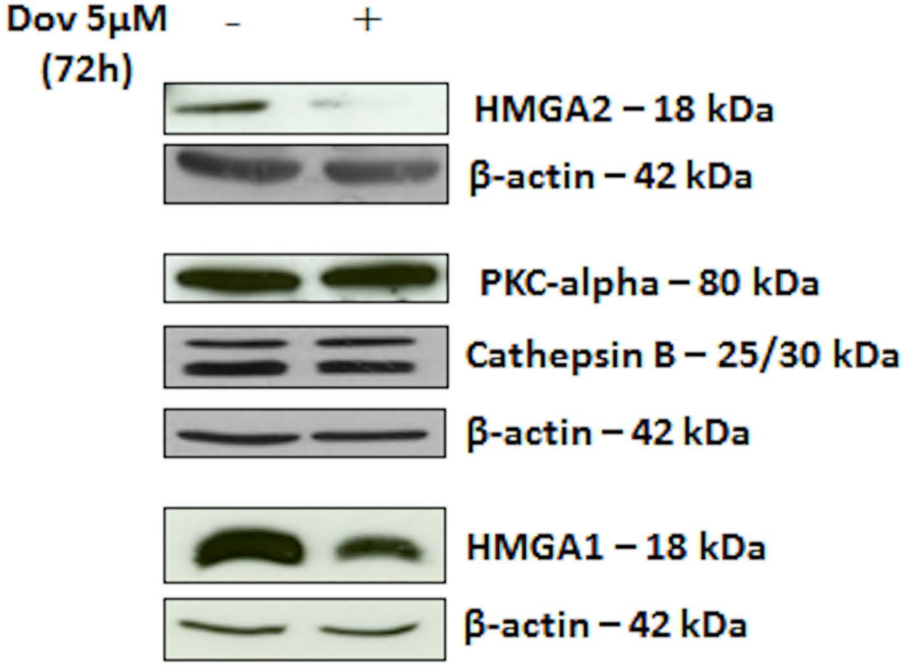

B.

U251- HMGA2 clone2

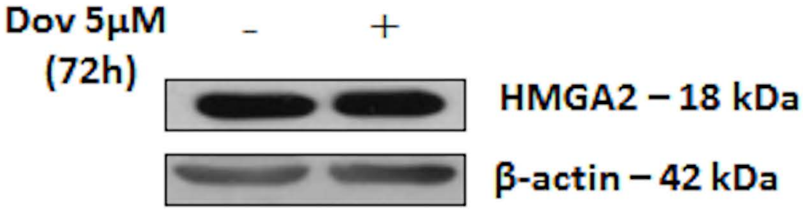

Supplement: Supplementary file 3 — Fig. S3. Dovitinib mediated specific down‐regulation of endogenous HMGA2. [file MOL2-11-1078-s003.pdf]
